# Caregiver and Adolescent Perspectives on Giving and Receiving Care After NonEmergency Surgery: A Qualitative Study

**DOI:** 10.1155/anrp/9344365

**Published:** 2025-04-02

**Authors:** Sydney E. S. Brown, Camila Costa, Alyssa Kelly, Sarah Oh, Daniel Clauw, Afton Hassett, Noelle E. Carlozzi

**Affiliations:** ^1^Department of Anesthesiology, University of Michigan, Ann Arbor, Michigan, USA; ^2^University of Michigan, Ann Arbor, Michigan, USA; ^3^Central Michigan University College of Medicine, Mount Pleasant, Michigan, USA; ^4^Department of Surgery, University of Michigan, Ann Arbor, Michigan, USA; ^5^Department of Physical Medicine & Rehabilitation, University of Michigan, Ann Arbor, Michigan, USA

**Keywords:** adolescent, caregiver experience, caregiving, qualitative research, recovery after surgery

## Abstract

**Objectives:** Over 1.5 million adolescents undergo surgery each year in the United States. While surgery is performed to improve pain and physical functioning, there may be temporary disability and an increased reliance on caregivers during recovery. Caregivers not accustomed to providing this level of care for adolescents used to greater independence may struggle. We sought to better understand the dyadic experience of at-home care for adolescent patients after nonemergency surgery.

**Methods:** We conducted semi-structured interviews with adolescent patients aged between12and 17 years and an associated caregiver, separately, 2 weeks following nonemergency surgery at a tertiary care pediatric hospital. Interviews were analyzed using latent manifest content analysis concurrent with data collection. Recruitment occurred until thematic saturation was reached.

**Results:** Semi-structured interviews were conducted among 31 adolescent-caregiver dyads. Sixteen caregivers and 12 adolescents described needing or providing help with activities of daily living (ADLs) and/or instrumental ADLs. Four themes emerged: (1) caregiver feelings of overwhelm, primarily among those helping with ADLs; (2) care activities described as something a “good caregiver” does contrasted with the more neutral way in which adolescents described needing help; (3) discrepancies between caregiver and adolescent perspectives regarding increased family interactions resulting from needing or providing care; and (4) the importance of peer friendships to adolescents throughout surgical recovery.

**Discussion:** Half of the adolescents and caregivers reported providing or requiring significant assistance with basic care needs after surgery. While some caregivers felt overwhelmed, others derived satisfaction from being a “good” caregiver and increased family time; adolescents felt more neutral about these interactions. Connections with friends (in-person or online) were helpful to adolescents. Results suggest that interventions directed toward improving caregiver support and helping them find positive aspects of caregiving, as well as encouraging adolescent connection with their friends may improve the perceived quality of recovery in this population.

## 1. Introduction

Over 1.5 million adolescents undergo nonemergency surgery each year in the United States, most of which are performed on an outpatient basis [1, 2]. While most surgeries are performed to improve pain and physical functioning, even surgeries considered to be minor may be painful and result in temporary disability and a new reliance on caregivers during recovery [3–5]. Caregivers not accustomed to providing this extensive postoperative care and adolescents used to greater independence may struggle. Furthermore, difficult family interactions, though normative during adolescence, may pose barriers to recovery just as they do in children with chronic health conditions; however, the content of such interactions among adolescent surgical patients and their caregivers has not been described [6, 7]. This is important because negative interactions can amplify acute postoperative pain [8], which could contribute to pain-related disability and chronic postsurgical pain. This risk is higher during adolescence than any other stage of childhood [3, 9, 10]. A better understanding of these experiences could lead to improved anticipatory guidance or interventions targeting various aspects of caregiving and the caregiver-adolescent relationship during recovery which could lead to improved pain and mental health outcomes.

We sought to better understand the caregiver experience of providing at-home care for an adolescent surgical patient, and the adolescent experience of receiving care after nonemergency surgery, in their own words. We were specifically interested in exploring what caregivers felt about their new caregiving responsibilities (overwhelmed vs. fulfilled), and what strategies they employed to cope with these difficulties. We were also interested in better understanding the interaction between adolescents and caregivers as care partners, as they navigated these challenges. Finally, we explored factors that adolescents believed helped them cope with recovery.

## 2. Materials and Methods

This study was approved by an institutional review board (HUM00213378; IRBMED, University of Michigan, Ann Arbor, MI); written consent, parental permission, and assent were obtained from each caregiver-adolescent dyad. The study was conducted in compliance with the Enhancing the QUAlity and Transparency Of healthcare Research (EQUATOR) network”s standards for reporting qualitative research [11].

### 2.1. Patient Recruitment

Patients aged 12–17 years having an outpatient nonemergency surgery at a tertiary care pediatric hospital were identified in the surgical schedule 1–5 days before surgery and targeted for recruitment for semi-structured interviews to take place 2–3 weeks after surgery with a primary caregiver. Enrollment included a range of surgical procedures to permit the exploration of caregiving experiences across a range of experiences.

Caregivers were contacted by phone by a study coordinator between June of 2022 and March of 2023. If both adolescent and caregiver were able and agreed to participate, consent, parental permission, and assent forms were sent to the caregiver via email. Interviews were scheduled 2–3 weeks after surgery because typically the most severe postsurgical symptoms requiring caregiver assistance should be resolved [12].

### 2.2. Data Collection

We developed and pilot tested an open-ended semi-structured interview guide (Supporting 1, Supporting 2) developed based on clinical expertise and a review of the literature [13–22]. We asked open-ended questions about giving and receiving care after surgery, such as the extent to which help completing daily activities—specifically activities of daily living (ADLs) and instrumental ADLS (IADLs)—and self-care following surgery were required and their own perspective on what it was like to give/receive care.

Patients were recruited until thematic saturation was reached. Generally, 15–20 interviews are sufficient to reach thematic saturation [23–26]. We planned to recruit at least 30 dyads because of the diversity in surgeries sampled. In addition to interviews, an electronic health record review was conducted identifying documented physical and psychological comorbidities, and previous surgeries within the past 2 years.

All interviews were conducted via Zoom. Adolescents and caregivers were interviewed separately. Interviews were conducted and transcribed by trained research staff that included a pediatric anesthesiologist (SB), a study coordinator (DH), a medical student (SO), and two bachelor's-level research assistants (GW, CC).

### 2.3. Data Coding and Analysis

First, transcribed interviews were analyzed by three reviewers (SB, AK, and CC) to obtain a holistic view of the data. We then used a deductive approach in which existing theories about giving and receiving care were applied to code creation, and novel codes were created as they emerged [27–31]. We used latent manifest content analysis, a method which analyzes surface and observable elements of content as well as the underlying, implicit meanings that are not directly expressed, to categorize data and extract themes endorsed by caregivers and adolescents [27, 32]. Specifically, we noted whether and how the participant described the experience of giving or receiving care and interactions with friends and family members. Discrepancies were discussed and adjudicated by review and discussion of the relevant interview text during weekly meetings [27]. Interview data were managed using NVivo software (QSR International, Melbourne, Australia).

Numeric data are presented using counts and percentages. We compared adolescent versus caregiver perspectives on some elements using Fisher's exact test.

## 3. Results

After interviewing 31 dyads, no new themes were generated from the interviews; therefore, it was deemed that thematic saturation had been reached, and data collection was stopped. Thirty-one adolescent-caregiver dyads completed separate interviews. Their characteristics are described in Table 1. Six caregivers and seven adolescents described helping or needing help with ADLs (Supporting Table 3). This was primarily reported among adolescents having orthopedic surgery (four of five surgeries) or tonsillectomy & adenoidectomy (T&A) surgery (three of six surgeries); however, some adolescents recovering from typically less painful procedures (e.g., cystoscopy with ureteral stents) also required help with ADLs (three of ninteen less painful surgeries). Participants cited needing or providing help with “everything,” including personal hygiene comprising bathing, hair brushing, and using the toilet, as well as getting in or out of a bed or chair.

Fifteen caregivers and nine adolescents described needing or providing help with IADLs, such as taking medications, meal preparation, and driving to or getting around school (Supporting Table 4). Assistance with IADLs was mentioned among four of five orthopedic surgery patients and four of six T&A patients. Nine of 19 adolescents recovering from surgeries typically associated with less painful recoveries also required help with IADLs.

### 3.1. Perspectives on Caregiving

Four themes emerged about the caregiver experience of providing postsurgical care to their teenage child and the adolescent's experience of receiving care: (1) caregiver feelings of being overwhelmed, primarily among those having to help with ADLs; (2) care activities performed because that is what a “good caregiver” does contrasted with the more neutral way in which adolescents described needing help, (3) discrepancies between caregiver and adolescent perspectives regarding increased family interactions resulting from needing or providing care during recovery; and (4) the importance of peer group friendships to adolescents throughout surgical recovery.

#### 3.1.1. Feeling Overwhelmed by Additional Responsibilities

All but one caregiver (five of six) who described helping with ADLs reported feeling overwhelmed or exhausted by their child's care needs after surgery (Table 2). One caregiver described how the discomfort her son experienced after surgery increased his reliance on her for basic needs like getting in and out of a chair and showering, some of which could have been avoided had they been better prepared by the surgeon about the postoperative course. Two caregivers likened providing care to their recovering adolescent child to having a much younger child again. For instance, one caregiver noted how,

“It's been more like having like a toddler again or something. She needed help with daily living things like getting to the bathroom, showering, meal preparation, all that stuff she needed pretty much everything done for her because she couldn't do it with one hand. (shoulder arthroscopy)”

Another described feeling exhausted caring for her son who was recovering from knee surgery, with his constant requests.

“So, he was just pretty much, you know, on the couch and you know, me having to help him up because he couldn't go to the bathroom by himself, he couldn't lift his leg by himself, he couldn't get out of bed by himself, so he needed a lot of help. He's always like, ‘Mom, I need this,' ‘Mom I need that,' so you know and like certain meals that he would make for himself, he can't do so I'm doing a lot more cooking and then a lot more, you know, getting up. So, I'm kind of tired. (knee arthroscopy)”

Two caregivers described contending with vomit and other bodily fluids during recovery, which they had not anticipated. One quote capturing the feelings of overwhelm came from a mother of an adolescent girl recovering from knee surgery.

“I felt like I had a baby again, you know, to help her get dressed and showered… I can't imagine having a child that you have to take care of all the time, you know, especially a young woman, and she starts her period, and then I'm trying to help her. I mean she just could hardly do anything on her own, literally in the shower, trying to get her on her crutches, get her leg out when you're not supposed to have any weight on your foot at all. (reconstruction dislocated patella)”

There was no significant difference in caregiver descriptions of feeling overwhelmed between adolescents who had received a prior surgery in the past 2 years and those who had not (80% vs. 86%, *p*=1.00).

#### 3.1.2. Being a Good Caregiver

Eight caregivers described deriving satisfaction from various caregiving activities because they were activities that a “good” caregiver would perform (Table 3). For instance, four caregivers described preparing meals for their adolescent children as something they felt a good caregiver should do after their child has surgery. One caregiver described how,

“You know, when someone comes out of surgery you offer that to them, ‘Oh can I get you something to drink,' ‘You want some pop,' ‘Do you want some juice,' You know, ‘What do you want to eat,' You know? I think that we do that automatically as her caregivers. (Tympanoplasty)”

Another said,

“I was pretty much making her food and getting her drinks and doing all of that for her, um, which normally she would do that for herself, so but it was not that she was incapable of doing it, but it was just sort of like, ‘I'm here, I can do that for you,' you know, ‘go rest,' you know, kind of a thing, where under normal circumstances I'd be like, ‘the kitchen's right there,' like, ‘go do it.' So, she got extra tender loving care just 'cause. (T&A)”

This was consistent with what other caregivers described.

Three caregivers described staying up late to care for their child. For instance, one caregiver said,

“I kept her downstairs with us until about what, 11 or 12 o'clock and then, finally I couldn”t stay up anymore and uh she said, ‘Mom it's okay, I can go to bed now,' I said, ‘Okay good let's go,' so I went up and tucked her in and she was fine. (Tympanoplasty)”

Two caregivers described making alternative family sleeping arrangements so their children would not be lonely. One described how,

“We all stayed pretty much at home with her the whole time. Her siblings and I… set up a bed out in the living room so that way she wouldn't feel like excluded or like alone. I knew she was going to be in a lot of pain but I didn't want her to feel lonely, so I made sure that she was there in the living room with us the whole time. (T&A)”

There were no significant differences in deriving satisfaction from caregiving between caregivers of adolescents who had surgery within the past 2 years and those who had not (80% vs. 67%, *p*=0.667).

#### 3.1.3. Adolescent Feelings About Receiving Care From Caregivers

Several caregivers reported that their child enjoyed being cared for, and not having to go to work, prepare meals, or do chores. However, four other caregivers reported that their child felt upset over their loss of independence, three of whom reported that their children didn't like asking for help. One caregiver reported that her child, “said a few times to me about being upset how much I've seen her naked now. You know, at 15 you don't really love it.” Another caregiver reported that she, “tried to offer assistance… but he didn't want it or need it.”

Most adolescents did not talk about their recovery as an opportunity to avoid chores or work, though two described how they were “just too lazy” to do activities like meal prep themselves while they were recovering from surgery. And while several caregivers talked openly about how they thought their child felt about needing help, only one adolescent talked about how he felt about the help he received during recovery. (10/31 caregivers vs. 1/31 adolescents, *p*=0.006). Instead, most used concrete language to describe the help they required (e.g., “I just needed help like going to the bathroom”; “[I needed help] making food for myself; “ “[I needed help] brushing my hair.” Only one boy talked about conflict that arose around needing help and his feelings surrounding that, saying, “Yeah, I guess mostly when people would… do nice gestures like, bring me food or something, and I knew I didn't want to eat, and then they would get mad at me for not eating… I just, I would get mad.”

#### 3.1.4. Perspectives on Family Time

Ten caregivers and six adolescents (seven total adolescent reports) reported increased family time during recovery (Table 4). Caregivers tended to describe this time positively (five of ten caregiver reports vs. one of seven adolescent reports) as opposed to neutrally (two of ten caregiver reports vs. three of seven adolescent reports) or negatively (three of ten caregiver reports vs. three of deven adolescent reports) compared with adolescent reports of family time though none of these differences were statistically significant. Four caregivers reported their family came together to help with caregiving. In one case, an adolescent's brother, “checked on him, made sure he was okay, see if he needed anything,” when he came home from work. The adolescent reported, “(I sat) in my room the whole time so I couldn't see (my family) much.” One caregiver described setting up her child's bed in the living room so she wouldn't feel excluded or lonely. Another caregiver reported fondly how her son helped care for his sister.

“I mean we're lucky. I have an 18-year-old that's been lifting weights for the last few years, and he would just scoop her up and carry like no problem… That first night, her brother carried her in and set her up on the couch. We have recliners and then [he] said, ‘You guys go to bed, I'll take care of her.' So, he slept next to her.”

These reports were corroborated by the adolescents; however, neither adolescents discussed how they felt about this extra time spent with family.

Three caregivers reported negative family interactions. Meals were a source of conflict for dyads 16 & 17. One girl (T&A) reported being angry at her mother for eating food in front of her she wasn't allowed, and one boy (T&A) reported being angry at his parent's persistence at trying to get him to eat (described in the section about adolescent feelings about receiving care). Finally, one father (T&A) reported that his child was more withdrawn as a result of taking opioid medication for pain, which “bugged the crap out of her mom,” who wanted to talk to her.

Adolescent report of family interactions tended to be more neutral such as, “I spent a lot more time with my parents because they were like helping me out and stuff,” or negative. One adolescent reported that she found the additional family time “overwhelming.” Only one adolescent reported enjoying this additional family time, despite also describing conflict with her mother over food, stating,

“Nope, I spent as much time as possible because I was actually home and normally, I don't spend as much time with my mom or my parents… not that I don't want to do that, but I just spent as much as time with my mom as possible (T&A).”

#### 3.1.5. Connections With Friends During Recovery

Twelve adolescents and 15 caregivers described how the adolescent's contact with friends during recovery (or lack thereof) was important to their recovery (Table 5). Three adolescents and one caregiver described friends visiting in-person during recovery. One adolescent even described how her friend who also had recently had a tonsillectomy came by to hang out, which she found helpful. Some interactions with friends took place via messaging or gaming. Two adolescents reported missing their friends because they were not able to use their phones because they had just had eye surgery or because their friends did not reach out.

A few adolescents and caregivers reported limiting contact with friends because of appearance or demeanor post-surgery. One caregiver reported that,

“Um well [her boyfriend] would come to her, which was nice, and then the other friends would come to her… but she wasn't that much fun, I mean, let's just be honest. And she was paranoid at first, too, with the boyfriend coming over because I don't know, that post-surgical breath situation with the tonsils was like a hot mess.”

Finally, one caregiver reported that she limited visitors to their home, including relatives, because “(her child) wouldn't be much company to anyone.”

## 4. Discussion

The purpose of this study was to gain a better understanding of the caregiver experience of caring for their adolescent child at home after nonemergency surgery, the adolescent experience of receiving care, and caregiver-adolescent interactions during recovery, each in the participant's own words. Half of the participating adolescents needed help with ADLs and/or IADLs for some time after surgery. Almost all caregivers helping their children with ADLs felt overwhelmed and exhausted. Some caregivers derived satisfaction from different elements of caregiving such as preparing meals or staying up late at night to care for their child. Furthermore, several caregivers noted increased family time which they mostly viewed positively, though it was not always clear how the adolescents felt about this additional time spent together as a family. Adolescents and caregivers also cited interactions with friends (or lack thereof) as important to recovery.

Our findings regarding feelings of overwhelm among caregivers providing help with ADLs are consistent with literature on caregiving of children receiving treatment for cancer and in caregivers of adult home care patients, which has found that the more strenuous and all-consuming care is, the more likely caregivers are to feel overwhelmed, [33–35]. This analysis is the only report to our knowledge among adolescents receiving surgical care from which recovery is expected in a relatively short timeframe.

The literature focusing on the positive aspects of caregiving for adolescent surgical patients is limited; however, one study in adult patients with dementia noted that, “the ability to find meaning in and derive gratification from the caregiving experience is associated with increased morale and a feeling of being more able to manage [36].” Another study reported that some caregivers experienced, “a sense of pride in carrying out care-related activities to a high standard,” and enjoyed the increased time spent with a loved one [37]. Future work should explore whether (1) better anticipatory guidance about postoperative care demands could ameliorate caregiver burden particularly when significant postoperative pain and disability are expected because of the surgery type or patient-specific risk factors, (2) whether interventions that help caregivers reframe caregiving activities in a positive light [38], either by highlighting the positive aspects of caregiving or providing suggestions on how to use recovery from surgery to create meaningful connections with the patient and other family members, could be used to enhance the well-being of caregivers in this population, leading to better care and recovery outcomes for the patient, and (3) whether caregivers with certain traits are more likely to exhibit resilience in response to increased caregiving demands, and whether these traits can be cultivated or shared with other caregivers.

Our findings regarding the positive influence of peer interactions on recovery quality is consistent with an adult study which found that patients with a larger more active social network experienced less intense pain and anxiety during the first week of recovery from surgery [39], and a second study of 36 pediatric surgical patients that found that increased peer caretaking interactions during surgical recovery (i.e. interactions with peers who have undergone the same surgery) was associated with lower levels of distress [40]. Future work could investigate how to encourage and facilitate positive interactions with peers, either in person or online, and measure whether this helps ameliorate pain and mental health symptoms experienced during recovery.

This study's primary limitation is that it is a qualitative study performed at a single tertiary care pediatric hospital located in the Midwest among largely white, well-educated participants. As a result, these data are likely not representative of all surgical recovery experiences. Furthermore, presurgical risk factors which may differentially affect recovery, such as family functioning, preoperative mental health symptoms, or pain, were not assessed. However, experiences reported by caregivers were consistent with those described in other adult and nonsurgical populations [33–37]. In addition, a strength of this study is the diversity of procedures and anesthetic types sampled. Past studies concerned with recovery in pediatric surgical patients have focused primarily on invasive surgeries known to be extremely painful such as for scoliosis or pectus repair; we have shown that significant issues related to caregiving and family relationships can arise after much less invasive procedures. Furthermore, we have also shown some ways in which caregivers derived satisfaction from their role, finding joy in helping their child and in increased family time. We also found that many adolescents felt that positive peer interactions made recovery easier. Qualitative research is, by definition, hypothesis generating, and this is the only study to our knowledge to query caregivers about the experience of caring for their previously healthy teen after minor surgery. Thus, the results of this work may inform future interventions to improve surgical recovery in adolescents.

## 5. Conclusions

Caregivers of adolescent surgical patients vary in the extent to which they find caregiving to be overwhelming and exhausting vs. satisfying which may be related to the extent to which their child requires help with ADLs after surgery as well as preexisting patient and surgical factors. Caregivers may derive satisfaction from performing duties associated with being a “good”” caregiver and increased family time; adolescents are more likely to speak of their care needs and increased family time factually. Adolescents may benefit from positive interactions from friends, either online or in person. Interventions directed toward improving caregiver support and helping them find meaning in caregiving and time spent with family, as well as encouraging connection between the adolescent and their friends in-person or online may improve the quality of recovery in this population.

## Figures and Tables

**Table 1:** Study population, surgery, and anesthetic characteristics.

**Patient Characteristics**	***N***	**%**

*Patient age (years)*		
12	5	16
13	3	10
14	6	19
15	6	19
16	6	19
17	5	16

Male patient	13	42

Male caregiver	5	16

*Patient race*		
White	24	71
Black	5	16
Asian	1	3
Biracial	1	3
Hispanic ethnicity	2	6

*Medical and surgical history*		
Previous surgery in past 2 years	10	32
Significant physical comorbidity^1^	5	16
Diagnosed psychological comorbidity^2^	8	26
Chronic pain history^3^	3	10

**Surgery type**	***N***	**%**

Otolaryngologic	7	23
Orthopedic	5	16
Plastic	6	19
Urologic	3	10
Ophthalmologic	2	6
General surgery	5	16
Other	3	10

^1^Seizure disorder, hypoplastic left heart, chronic kidney disease s/p kidney transplant, obstructive sleep apnea, leukemia, IgA deficiency, eosinophilic esophagitis, femoral epiphyseal dysplasia.

^2^Anxiety, depression, attention deficit hyperactivity disorder, autism.

^3^Migraine, chronic abdominal pain.

**Table 2:** Feeling overwhelmed by new caregiving responsibilities.

Endorsement rates	Example quotation
5/6 caregivers who assisted their adolescent children with ADLs 04c, 05c, 14c, 23c, 29c	• So he was just pretty much, you know, on the couch and you know, me having to help him up because he couldn't go to the bathroom by himself, he couldn't lift his leg by himself, he couldn't get out of bed by himself, so he needed a lot of help. He's always like “mom, i need this,” “mom I need that” so you know and like certain meals that he would make for himself, he can't do so I'm doing a lot more cooking and then a lot more, you know, getting up. So I'm kind of tired. (04-left knee arthroscopy of osteochondritis dissecans lesion)
	• It's been more like having like a toddler again or something. She needed help with daily living things like getting to the bathroom, showering, meal preparation, all that stuff she needed pretty much everything done for her because she couldn't do it with one hand. (05-shoulder arthroscopy)
	• I had to help her, uh, ‘cause she didn't feel like she could stand in the shower. So i just put her in the tub because i could manage it better. And i was covered in vomit. (14-tonsillectomy and adenoidectomy)
	• I felt like i had a baby again, you know, to help her get dressed and showered… I can't imagine having a child that you have to take care of all the time, you know, especially a young woman, and she starts her period, and then i'm trying to help her. I mean she just could hardly do anything on her own, literally in the shower, trying to get her on her crutches, get her leg out when you're not supposed to have any weight on your foot at all. (23-reconstruction dislocated patella)
	• He had trouble getting in and out of like, sitting up and down from his chair. Sometimes he needed a little help to lower himself into his chair. I had to do more like setting things up for him so that he could get to them and didn't have to like stand and move around a whole lot while he was in there. He took this a shower in a different bathroom, so he could sit down in my shower because it's got a bench. (29-cystoscopy with ureteral stents)

**Table 3:** Caregiving activities performed because that's what a “good” caregiver does.

Example Quotation
*Preparing food*
• You know, when someone comes out of surgery you offer that to them, “oh can I get you something to drink,” “you want some pop,” “do you want some juice,” you know, “what do you want to eat” you know? I think that we do that automatically as her caregivers. (02-tympanoplasty)
• I was pretty much making her food and getting her drinks and doing all of that for her, um, which normally she would do that for herself, so but it was not that she was incapable of doing it, but it was just sort of like “I'm here, I can do that for you,” you know, “go rest,” you know, kind of a thing, where under normal circumstances I'd be like, “the kitchen's right there,” like, “go do it.” so she got extra tender loving care just 'cause. (14-tonsillectomy & adenoidectomy)
• Um! there was once when he did get upset with me, I think, just like a little bit angry at me for asking him. Can I get you this? Can I get you that? Just because i think he felt like nothing's helping, and, you know, stop asking me. Nothing works, nothing helps. (16-tonsillectomy and adenoidectomy)
• I catered to him a lot at first because I'm his mom, so i just brought him food and stuff. But I think he probably could have handled it himself if he had, if he needed to, I just didn't make him. (21-laparoscopic cholecystectomy)

*Staying up late with their child*
• You get used to (being woken up at night) after the first (baby) you realize you're never going to sleep. (01-excision preauricular cyst)
• I kept her downstairs with us until about what, 11 or 12 o'clock and then, finally I couldn't stay up anymore and uh she said “mom it's okay, I can go to bed now” I said “okay good let's go”, so I went up and tucked her in and she was fine. (02-tympanoplasty)
• We all stayed pretty much at home with her the whole time. Her siblings and I… set up a bed out in the living room so that way she wouldn't feel like excluded or like alone. I knew she was going to be in a lot of pain but I didn't want her to feel lonely, so I made sure that she was there in the living room with us the whole time. (13-tonsillectomy & adenoidectomy)
• Before she was able to manage herself in the middle of the night, like if she woke up, she would need like just that moral support, that sort of like company to get through it. (14-itonsillectomy & adenoidectomy)
• I mean we're lucky I have an 18 year old that's been lifting weights for the last few years, and he would just scoop her up and carry like no problem… I mean, we come in with her that first night, and her brother carried her in and set her up on the couch. We have recliners and then said, you guys go to bed, I'll take care of her. So he slept next to her. (23-reconstruction dislocated patella)

*Other*
• And just we had to be extra careful with going to all the amusement parks and that, to make sure that we had it covered with sunscreen and make sure she was wearing a hat and things like that. (10-nevus excision)
• I tried to offer assistance because his bedroom is in the basement, so it's downstairs, um, but he didn't want it or need it (21-laparoscopic cholecystectomy)

**Table 4:** Perspectives on family time—example quotations.

**Caregiver perspective: (10/31) 02c, 07c, 09c, 13c, 14c, 16c, 17c, 23c, 26c, 29c**

*Positive interactions: 5/10*
• She was very lovey dovey like “oh I love you mom,” “can you hold my hand mom,” “don't go anywhere mom I don't want you to leave me.” other than that no, she enjoyed being home. I think she kind of enjoyed the break really, she's had a busy summer. (02-tympanoplasty)
• He had stayed at my parents for a couple days, because I had to work and they kept in close contact and he just you know laid on the couch a lot and like he said, they spoiled him with bringing him food. (07-enucleation of cyst)
• Both of us took really good care of him, so he was able to spend time with us, you know. His brother was at work, but when he came home he still, you know, checked on him, made sure he was okay, see if he needed anything, yeah. (09-chalazion removal)
• We all stayed pretty much at home with her the whole time. Her siblings and I… set up a bed out in the living room so that way she wouldn't feel like excluded or like alone. I knew she was going to be in a lot of pain but I didn't want her to feel lonely, so I made sure that she was there in the living room with us the whole time. (13-tonsillectomy & adenoidectomy)
• I mean we're lucky i have an 18 year old that's been lifting weights for the last few years, and he would just scoop her up and carry like no problem… I mean, we come in with her that first night, and her brother carried her in and set her up on the couch. We have recliners and then said, you guys go to bed, i'll take care of her. So he slept next to her. (23-reconstruction dislocated patella)

*Neutral interactions: 2/10*
• She and I were like attached to the hip for like, you know, 12 days or whatever, so we're like super tight. Everybody else was um, you know, her sisters would hang out with her when they were home, but they still wanted to go do their thing and they were still going to work and doing what they do. And then, when dad was home from work, he'd hang out as well, too, but um, you know, she and I were like, yeah, BFF's. For better or for worse. (14-tonsillectomy and adenoidectomy)
• Those 3 and a half days it was, he was pretty, you know much a hermit in his room, and I would go hang out with him. (29-cystoscopy with ureteral stents)

*Negative interactions: 3/10*
• Um! there was once when he did get upset with me, i think, just like a little bit angry at me for asking him. Can I get you this? Can I get you that? Just because I think he felt like nothing's helping, and, you know, stop asking me. Nothing works, nothing helps. (16-tonsillectomy and adenoidectomy)
• She was sad because she couldn't eat food, and she was mad at me because I put her through that surgery. (17-tonsillectomy and adenoidectomy)
When she went on like the oxycodone like it may have (made her) a bit more withdrawn, and may be sort of less engaged, like we'd have to like sort of draw her out a little bit there… and I think that… when you're not speaking as much it's just you seem more distant, so. 9. (26-tonsillectomy and adenoidectomy)

**Adolescent perspective: (7/31) 01a, 03a, 05a, 07a, 16a, 17a x2**

*Positive interactions: 5/10*
• Nope, I spent as much time as possible because I was actually home and normally I don't spend as much time with my mom or my parents, not that I don't want to do that, but i just spent as much as time with my mom as possible. (17-tonsillectomy & adenoidectomy)

*Neutral interactions: 3/7*
• You know I had more time to hang out with (my family) because I couldn't really do anything else. (03-wrist ganglion cyst excision)
• Um well, I spent a lot more time with my parents because they were like helping me out and stuff. (05-shoulder arthroscopy with open biceps tenodesis)
No, not with my friends. I Went with my grandparents so I increased time with them. (07-enucleation of cyst)

*Negative interactions: 3/7*
• My time with my family was overwhelming. (01-excision preauricular cyst)
• I guess mostly when people would do nice gestures like, bring me food or something and I knew I didn't want to eat, then they got mad at me for not eating, and then I would get mad. (16-tonsillectomy & adenoidectomy)
• I was very angry because I was, I was mad at her because I couldn't eat anything and she was eating my favorite things right in front of me and I was like so mad. (17-Tonsillectomy and adenoidectomy)

**Table 5:** Connections with friends during recovery—example quotations.

**Caregiver Perspective: (15/31) (04c, 05c, 06c, 08c, 11c, 12c, 14c, 16c, 17c, 18c, 21c, 23c, 24c, 26c, 30c)**

*Positive experiences connecting with friends during recovery*
• Some friends, you know came over to see her and hang out, just watch some TV and movies together so that's helpful. (05-shoulder arthroscopy with open biceps tenodesis)
• Actually, the next day, one of her best friends came over and they watched movies. (06-knee arthroscopy with chondroplasty)
• He could still talk to his friends online and stuff, and though they are friends online, they're also his school friends, so he kept up with his school friends through online gaming. (21-toenail excision)
• (She was able to keep up with friends by) you know messaging them and that sort of thing. (26-tonsillectomy & adenoidectomy)

*Difficulties connecting with friends during recovery*
• And she likes her routine too, so I think just like the break in routine, not being able to go in (to school) and see her friends. (08-fractional CO2 laser for scar revision)
• (He's) pretty social. I would kind of call him a social butterfly, so it's kind of annoying that he can't go to school. (12-spinal tap)
• Um well (her boyfriend) would come to her, which was nice, and then the other friends would come to her… but she wasn't that much fun, I mean, let's just be honest. And she was paranoid at first, too, but the boyfriend coming over because I don't know, that post-surgical breath situation with the tonsils was like a hot mess. (14-tonsillectomy & adenoidectomy)
• She couldn't eat, she couldn't see her friends. (17-tonsillectomy & adenoidectomy)
• Yeah, I mean, we didn't really have anyone over, you know, to visit and stuff just because, you know, she wouldn't be much company to anyone. (23)
• It was hard not being able to see because of eye surgery, and not being able to use phone/devices. (24-eye surgery)
• I mean, she, she wasn't going to school. So, you know there's a bit less interaction there. She didn't go to fencing. I Know she was in touch with her friends. (26-tonsillectomy & adenoidectomy)

*Neutral experience connecting with friends during recovery*
• She's pretty introverted, so I would say she missed youth group, but as far as she's not, she wasn't that upset about missing it, so. (18-mass excision-left lower back)0

**Adolescent perspective (12/31) 04a, 05a, 06a, 07a, 08a, 09a, 10a, 11a, 12a, 14a, 16a, 19a**

*Positive experiences connecting with friends during recovery*
• I can still play with my friends like online (04-left knee arthroscopy of osteochondritis dissecans lesion)
• I just wasn't able to like go places for like a week or two. They still came to me. (11-right cervical lymph node excision)
• But like my other friend got her tonsils out a week before me so she came and sat by. (14-tonsillectomy & adenoidectomy)

*Difficulties connecting with friends during recovery*
• The part that's been most frustrating for me is just like soccer because my team just started practicing for the season. And they were out practicing, running the beep test which sounds awful, but like obviously i want to be doing it because I'm just sitting around and kind of like doing nothing (06-knee arthroscopy with chondroplasty)
• We were supposed to go out to one of our friend's but couldn't. Also I couldn't listen to music because i couldn't look at my phone really. Playing a game, i couldn't play any games. (09-eye surgery)
• I like seeing people going out and like walking and stuff because my sister likes to walk around outside and stuff, um and like at the time I just couldn't. (13-tonsillectomy & adenoidectomy)
• So, another thing with the surgery is I couldn't really talk for like a week and a half. I was like mumbling. I couldn't, really, I don't know there was just so much saliva in my mouth. So, I didn't really go out anywhere or see friends for at least like 2 weeks after surgery… it was right before college kind of happened, so I didn't see my friends before they went off to college for the most part. (16)
Um it only really affected my social life when I didn't go to school when I was recovering. (19)

## Data Availability

The data that support the findings of this study are available on request from the corresponding author. The data are not publicly available because of privacy or ethical restrictions.

## Supporting Information

Supporting Information 1 Supporting 1: Patient interview guide.

Supporting Information 2 Supporting 2: Proxy interview guide.

Supporting Information 3 Supporting 3: Labor associated with caregiving-activities of daily living.

Supporting Information 4 Supporting 4: Labor associated with caregiving-instrumental activities of daily living.

Supporting Information 5 Supporting 5: SRQR checklist.
