# Supplementary material for: Caregiver and Adolescent Perspectives on Giving and Receiving Care After NonEmergency Surgery: A Qualitative Study
Source: Anesthesiol Res Pract. 2025 Apr 2;2025:9344365. doi: 10.1155/anrp/9344365 (PMC11981702; doi:10.1155/anrp/9344365)
Supplement: Supporting Information 2 — Supporting 2: Proxy interview guide. [file 9344365.f2.docx]

Identification of Post-Operative Symptoms Important to Adolescents and Their Caregivers

Manual of Procedures: Phase 1 – Semi-Structured Interviews

**DRAFT: Proxy/Parent Interview Guide**

Opening Statement

Thank you for agreeing to participate in this project. I’m XXX and I will be your interviewer for this discussion. I work in the Department of Anesthesiology at the University of Michigan. The purpose of this interview is to learn more about common symptoms and other problems adolescents experience after “outpatient” surgery (this is where the adolescent goes home the same day). The best way to learn about these things is to talk those individuals who have recently gone through this experience. Once we better understand what symptoms and other problems bother adolescents’ experiences, we will be able to study ways to improve these symptoms in children we take care of in the future.

We’ve asked you to participate in this interview so that we can talk with you about your child’s experiences. As we go through the questions, it will be really important for you to say what you think, and to tell us what recovery from surgery has been like for YOUR CHILD. There are no right or wrong answers. If you would like to add to an idea, or if you have an idea that’s different from what you think someone else might think or feel, or even different from the questions we are asking you, please tell us! We are here to learn from you.

We will be audio record this session to be sure we don’t miss anything you say. This survey will be confidential, meaning that we will not tell anyone about what you said – ever -- including your doctors or your parents/family, UNLESS you tell us you wish to harm yourself or other people.

In order to help us keep things confidential, we will create a study ID that will allow us to link details from your medical history and surgery to the recording, but the recording itself will not include your name or other identifying information. The file containing the link between the study ID and your personal information will be kept in a secure location that only approved and trained members of the study team will have access to. If you make a mistake and mention your name or something else while we are recording, we will remove that information from the transcripts.

Please speak up and talk clearly so that we can understand what you are saying. We only have 30 minutes or so, so it will be my job to keep us moving along. If you don’t understand a question, please let me know, and I will clarify what we mean to the best of my ability.

**I. Introduction & Ice-Breaker**

For example: *“Why don’t we start with introductions. Can you tell me a little bit about your child? What do they like to do for fun? Can you tell me why you and/or your child decided to participate in the interview?”*

**II. Free List Exercise on Quality of Life**

Sample Questions:

- *Please tell me about your child’s recent surgery.*
- *How did your child feel when they woke up from surgery?*
- *How did your child feel when they got home from the hospital after having surgery?*
- *What types of symptoms did they have?*
- *What kinds of things/what areas of their life have been affected because of this surgery?*
- *How did their recovery from surgery impact:*
  - *Your child*’s *social life*

PROBES

- - - *Like time spent with friends*
    - *Or time spent with family?*
  - *The things they like to do for fun? (Can refer back to what the subject said their child liked to do for fun at the beginning of the interview)*
  - *Your child’s mood or emotions?*

PROBES

- - - *How anxious or worried your child feels*
    - *How angry your child feels*
    - *How frustrated your child feels*
    - *How sad your child feels*
  - *How your child is able to participate in school?*

PROBES

- - - *Getting to or around school during the day*
    - *In class*
    - *After school*
  - *How your child feels physically*

PROBES

- - - - *Such as how tired they feel*
      - *How well they are able to sleep at night*
      - *How much pain they have*
      - *How stiff their body feels*
      - *How well they are able to get around, for instance getting out of bed or a chair, walking around the house, or getting around at school*
      - *How nauseated they feel, or whether their appetite is the same as it was before surgery*
      - *Whether they feel short of breath, or like they are having trouble breathing*
      - *Whether they are able to go to the bathroom normally*
  - *How much help they have needed to do your usual activities*

PROBES

- *Getting food for themselves*
- *Going to the bathroom*
- *Taking a shower*
- *Getting to school and getting around school*
- *Homework*
- *Chores*
- *After school jobs (if applicable)*
- *After school*

If additional questions are needed to elicit responses:

*Do these symptoms come and go during the day?*

*Are these symptoms worse during the day or at night?*

*How long do these symptoms last?*

*How long did it take for your child to be able to return to their old routine?*

Additional Probes (if not discussed previously):

1. *Emotions (moods and feelings)?*
2. *Physical abilities and limitations (sleep difficulties and muscle tension)?*
3. *Social interactions (relationships with family, friends, peers, coworkers)?*
4. *Your ability to do things for yourself and participate in your usual activities like school or work?*

NOTE: Emotional, Physical, and Social Health, and Independence should be discussed.

**III. Closure**

*“Thinking back to our discussion, is there anything else that I didn’t ask you about that you think would be helpful to share with me about your child’s recovery after surgery?*

*“Thank you again for your participation. We can’t do this work without you because no one else can tell us what issues are most important to adolescents as they recover from surgery. The survey that we are creating will be very important in better understanding surgical recovery, and ultimately in improving the care children receive before, during, and after they have surgery.”*
