# Supplementary material for: Caregiver and Adolescent Perspectives on Giving and Receiving Care After NonEmergency Surgery: A Qualitative Study
Source: Anesthesiol Res Pract. 2025 Apr 2;2025:9344365. doi: 10.1155/anrp/9344365 (PMC11981702; doi:10.1155/anrp/9344365)
Supplement: Supporting Information 3 — Supporting 3: Labor associated with caregiving-activities of daily living. [file 9344365.f3.docx]

**Supplemental Table 3. Labor Associated with Caregiving – Activities of Daily Living**

| Endorsement Rates | Exemplar Quotations |
| --- | --- |
| Caregiver  6/31  04c, 05c, 14c, 23c, 29c, 30c | *Needing Help with “Everything”*   - I felt like I had a baby again, you know, to help her get dressed and showered... I can't imagine having a child that you have to take care of all the time, you know, especially a young woman, and she starts her period, and then I'm trying to help her. I mean she just could hardly do anything on her own, literally in the shower, trying to get her on her crutches, get her leg out when you're not supposed to have any weight on your foot at all. (23 – reconstruction dislocated patella) - So he was just pretty much, you know, on the couch and you know, me having to help him up because he couldn't go to the bathroom by himself, he couldn't lift his leg by himself, he couldn't get out of bed by himself, so he needed a lot of help. (04 – knee arthroscopy, OCD lesion) - It's been more like having like a toddler again or something. She needed help with daily living things like getting to the bathroom, showering, meal preparation, all that stuff she needed pretty much everything done for her because she couldn't do it with one hand. (05 – shoulder arthroscopy)   *Standing and Walking*   - He had trouble getting in and out of like, sitting up and down from his chair. Sometimes he needed a little help to lower himself into his chair. I had to do more like setting things up for him so that he could get to them and didn't have to like stand and move around a whole lot while he was in there. He took this a shower in a different bathroom, so he could sit down in my shower because it's got a bench. (29 – cystoscopy with ureteral stents)   *Bathing*   - I had to help her, uh, 'cause she didn't feel like she could stand in the shower. So I just put her in the tub because I could manage it better. And I was covered in vomit. (14 – tonsillectomy and adenoidectomy) - So the first few weeks I was doing the hair washing and we did find somebody to help her. (30 – clavicle hardware removal) |
| Adolescent  7/31  02a, 05a, 06a, 11a, 13a, 16a, 23a | *Needing Help with “Everything”*   - It’s just it's pretty hard to go to like to go to the bathroom in general, and then, like, with your leg like that, she means and locked in extension. It's hard to sit down on the toilet and stuff. And then the first like 3 days, my brother or my dad carried me to the bathroom. I definitely needed like help getting into the shower and all that I had a seat for that. Yeah, I still sometimes need help to like, get up like, sit on like higher places like my bed and stuff. Sometimes I can swing it back up, but not always. (23 – reconstruction dislocated patella) - Even just like day-to-day things like getting dressed and showering and stuff are like definitely harder. (05 – shoulder arthroscopy)   *Personal Hygiene*   - Right after the surgery I just needed help like going to the bathroom. My mom would make sure I didn't fall because we had to stop by the bathroom right before we got home. And then she washed my hair one time and then I was good. (02 – tympanoplasty) - I had to call my mom so like she could help me go to the bathroom and stuff like that because I had like that huge thing on my leg and like I couldn't get like my pants over it when I had to pull them down. [And I needed help] like getting off the couch or like in a chair. (06 – knee arthroscopy, chondroplasty) - Brushing my hair because I really couldn't move (11 – cervical lymph node excision)   *Standing and Walking*   - Walking or just like regular like getting down the stairs and stuff. (13 – tonsillectomy and adenoidectomy)   *Eating*   - My parents would feed me, or something like that for the first few days. (16 – tonsillectomy and adenoidectomy) |
