# Supplementary material for: Caregiver and Adolescent Perspectives on Giving and Receiving Care After NonEmergency Surgery: A Qualitative Study
Source: Anesthesiol Res Pract. 2025 Apr 2;2025:9344365. doi: 10.1155/anrp/9344365 (PMC11981702; doi:10.1155/anrp/9344365)
Supplement: Supporting Information 4 — Supporting 4: Labor associated with caregiving-instrumental activities of daily living. [file 9344365.f4.docx]

**Supplemental Table 4. Labor Associated with Caregiving – Instrumental Activities of Daily Living**

| Domain | Example Quotation |
| --- | --- |
|  |  |
| Caregivers  15/31  01c, 02c, 03c, 04c, 05c, 07c, 09c, 10c, 13c, 14c, 16c, 21c, 23c, 24c  30c    *Post surgical Care*  01c, 02c, 05c, 07c, 10c, 24c  *Meal Preparation*  01c, 02c, 03c, 04c, 05c, 07c, 09c, 13c, 14c, 16c, 21c  *Getting Around, Driving, Carrying Things*  04c, 05c, 23c, 30c | **Post-surgical Care**   - So she kept waking up and, of course, she would wake me up. You get used to it after the first one you realize you're never going to sleep. (01 – Excision preauricular cyst) - Just by having to put ear drops in her ears which she can't stand. (02 – tympanoplasty) - I was waking her up for pain medicine (05 – shoulder arthroscopy) - Watching the timing of his medicine so that it was timed out properly was the only thing. (07 – cyst enucleation) - I helped her change the bandage. And just we had to be extra careful with going [outside], to make sure that we had it covered with sunscreen and make sure she was wearing a hat and things like that. (10 – nevus excision) - She couldn’t open her eyeballs a lot. She was a little bit of a princess wanting to get the ice packs changed… like ‘Oh, help me. I need this. Help me,’ kind of thing.” (24 – strabismus surgery)   **Meal Preparation**   - I don't know if we assisted her in eating, but we did adjust for her limitations. So we gave her like hand like finger foods, instead of like trying to have her use a spoon or fork the first day and then the following day. (01 - excision preauricular cyst) - You know, when someone comes out of surgery you offer that to them. "oh can I get you something to drink," "you want some pop," "do you want some juice," you know," What do you want to eat" You know. I think that we do that automatically as You know, her caregivers, caretaker. (02 – tympanoplasty) - And I always, you know, somebody is always making food for him but other than that. (03 – wrist ganglion cyst excision) - So you know he's always like "Mom, I need this", "Mom I need that" so you know and like certain meals that he would make for himself, he can't do so I'm doing a lot more cooking and then a lot more, you know, getting up. So I'm kind of tired. (04 - Left knee arthroscopy of osteochondritis dessicans lesion) - She needed help with… meal preparation… because she couldn't do it with one hand. (05 - Shoulder arthroscopy with open biceps tenodesis) - Just talking it out with him on what he could or couldn't eat, what he felt comfortable doing, so we'd have the right dinners and food out for him. (07 - Enucleation of cyst) - I cooked every day, so he was able to eat, you know, food and stuff. (09 - Chalazion removal) - Just giving her food and like making sure she was eating and trying to eat something. (13 - Tonsillectomy & adenoidectomy) - I was pretty much making her food and getting her her drinks and doing all of that for her, um, which normally she would do that for herself, so but it was not that she was incapable of doing it, but it was just sort of like "I'm here, I can do that for you," you know, "go rest," you know, kind of a thing, where under normal circumstances I'd be like, "the kitchen's right there," like, "go do it." So she got extra tender loving care just 'cause. (14 -- Tonsillectomy & adenoidectomy) - Um! There was once when he did get upset with me, I think, just like a little bit angry at me for asking him. Can I get you this? Can I get you that? Just because I think he felt like nothing's helping, and, you know, stop asking me. Nothing works, nothing helps. (16 - tonsillectomy and adenoidectomy) - I catered to him a lot at first because I’m his mom, so I just brought him food and stuff. But I think he probably could have handled it himself if he had, if he needed to, I just didn’t make him. (21 - Laparoscopic cholecystectomy)   **Getting Around, Driving, Carrying Things**   - Of course he can’t carry anything like with the crutches. (04 - knee arthroscopy, OCD lesion) - She needs help in order to do most things, and like not being able to drive right now. (05 – shoulder arthroscopy) - School is really hard, like I had to drive her up there… and she's going have to have someone carry her bag (23 – reconstruction of patella) - So just kind of then having she didn't really want to go and ask somebody to help her carry her backpack (30 – hardware removal, clavicle) |
| Adolescent  10/31  07a, 09a, 03a, 05a, 09a, 13a, 16a, 17a, 23a, 29a  *Post surgical Care*  07a, 09a, 13a  *Meal Preparation*  03a, 05a, 09a, 16a, 17a, 29a | **Post-surgical Care**   - - Like I had to take [my medications] at 2:00 in the morning and they had to wake me up so they had to get up at 2:00 to come wake me up. But over time, I got used to it and did it myself. (07 – cyst enucleation) - Putting the medicine in my eye. (09 – chalazion removal) - I was waking her up because I was told not to let the medicine lapse because if I did that, then it could be more painful and harder to get the pain under control. (13 – tonsillectomy & adenoidectomy)   **Meal Preparation**   - [I no longer had] the ability to make myself food whenever I want. (03 -- wrist ganglion cyst excision) - [I needed help with] just like making food, like making meals. (05 -- Shoulder arthroscopy with open biceps tenodesis) - Really for everything else, it was just like laziness, like them just bringing me food, but like I could do everything else myself. (07 -- Enucleation of cyst) - Um, making food. Yeah, probably making food. (09 -- Chalazion removal) - I guess mostly when people would do nice gestures like, bring me food or something and I knew I didn't want to eat, then they got mad at me for not eating, and then I would get mad. (16 - Tonsillectomy & adenoidectomy) - I was very hangry because I was, I was mad at her because I couldn’t eat anything and she was eating my favorite things right in front of me and I was like so mad. (17 – Tonsillectomy and adenoidectomy) - The only thing it really was is making food because I just didn’t feel like getting up, I felt like staying in bed. (17 - Tonsillectomy & adenoidectomy) - They always brought me food. (23 – reconstruction dislocated patella) - I couldn't cook for myself, because I couldn't like walk into the kitchen and stand up for a long time. (29 – Cystoscopy with ureteral stents) |
